# Water temperature induces jaw deformity and bone morphogenetic proteins (BMPs) gene expression in golden pompano *Trachinotus ovatus* larvae

**DOI:** 10.1186/s40064-016-3142-0

**Published:** 2016-09-02

**Authors:** Zhenhua Ma, Nan Zhang, Jian G. Qin, Mingjun Fu, Shigui Jiang

**Affiliations:** 1 South China Sea Fisheries Research Institute, Chinese Academy of Fishery Sciences, Guangzhou, 510300 China; 2 Key Laboratory of South China Sea Fishery Resources Exploitation and Utilization, Ministry of Agriculture, Guangzhou, 510300 China; 3 School of Biological Sciences, Flinders University, GPO Box 2100, Adelaide, SA 5001 Australia

**Keywords:** Temperature, Jaw deformity, Bone morphogenetic proteins, Golden pompano *Trachinotus ovatus*

## Abstract

Golden pompano *Trachinotus ovatus* larvae were kept at 26, 29 and 33 °C for 15 days from 3-day post hatching (DPH) to 18 DPH to test temperature-dependent growth and jaw malformation. The growth, survival, jaw deformity and the gene expressions of bone morphogenetic proteins (BMPs) were used as criteria to examine the fish response to temperature manipulation. The growth rate of fish at 29 or 33 °C was significantly faster than fish at 26 °C, while fish survival at 29 °C was significantly higher than fish at 33 °C. Jaw deformity was significantly affected by water temperature. The highest jaw deformity occurred on fish at 33 °C, and the lowest jaw deformity was at 26 °C. The expressions of all BMP genes except BMP10 were significantly affected by water temperature. The highest gene expression of BMP2 was on fish at 29 °C, and the lowest expression was at 33 °C. For the BMP4 gene, the highest and lowest expressions were found on fish at 33 and 26 °C, respectively. The present study indicates that jaw deformity of golden pompano larvae increases with increasing temperature, and the gene expression of BMP4 proteins coincides with high jaw deformity and water temperature elevation.

## Background

Temperature is a primary factor affecting early development of fish larvae and can regulate fish metabolism and feeding behavior during fish ontogeny (Kestemont and Baras 2001; Ma 2014). Furthermore, studies have demonstrated that the inappropriate range of temperature can lead to high mortality and body malformation on fish larvae (Lein et al. 1997; Ørnsrud et al. 2004; Ludwig and Lochmann 2009).

Jaw and skeletal malformations are often associated with poor growth and low survival of fish larvae and are a major bottleneck for efficient production of marine fish juveniles for aquaculture (Koumoundouros 2010; Boglione et al. 2013a, b). Jaw malformation can reduce fish survival and devalue fish quality on market (Barahona-Fernandes 1982; Cobcroft et al. 2004; Ma et al. 2014c). Jaw and skeletal malformations have been frequently observed on fish species in aquaculture such as striped trumpeter *Latris lineata* (Cobcroft et al. 2012), gilthead sea bream *Sparus aurata* (Andrades et al. 1996; Prestinicola et al. 2013), and yellowtail kingfish *Seriola lalandi* (Cobcroft et al. 2004). Lein et al. (1997) demonstrated that the increase of water temperature can induce jaw deformities. Under suboptimal temperatures, significant deformities of gill-cover and skeleton occur on gilthead seabream *Sparus aurata* (Georgakopoulou et al. 2010) and cranial deformities on European sea bass *Dicentrarchus labrax* (Georgakopoulou et al. 2007). In golden pompano *Trachinotus ovatus*, over 33 % of fish population exhibited at least one type of malformation during the larval period (Ma et al. 2014c; Zheng et al. 2014), but it is unclear if temperature leads to jaw deformities in this species. Therefore, it is necessary to explore the relationship between temperature and jaw deformity during early ontogeny of golden pompano larvae.

Skeletogenesis is a process of cell differentiation and proliferation in chondrocytes, osteoblasts, osteocytes and osteoclasts, and these cells determine the size, shape and mineral composition of bone structure (Nijweide et al. 1986; Karsenty and Wagner 2002; Phan et al. 2004). The gene expression during skeletogenesis is affected by both genetic and abiotic factors (Boglione et al. 2013a, b). Therefore, unveiling gene networks may provide insights into the potential mechanisms of skeletal malformations. The abiotic and biotic factors could induce skeleton deformity, while the gene expression drives the functional change of organs mediated by environmental factors.

In vertebrates, bone morphogenetic proteins (BMPs) control bone formation at different cell developmental states (e.g., stem cells, proliferative and hypertrophic chondrocytes, maturing osteoblast) (Hogan 1996a, b; Alaee et al. 2014; Windhausen et al. 2015). BMPs are genetically conserved in the animal kingdom, and their biological importance is reflected through functional and structural redundancy of different BMPs in a single species (Razdorov and Vukicevic 2012). For instance, BMPs 1, 2 and 3 can stimulate osteoblast, and play an important role in bone fracture healing (Grgurevic et al. 2011). BMPs 2, 4 and 6 are involved in skeletogenesis, especially in differentiation of chondrocytes to form cartilage, and both differentiation and maturation of the chondrocytes in the osteoblastic lineage will give a rise to bone formation (Rickard et al. 1994; Minina et al. 2001; Canalis et al. 2003; Wan and Cao 2005). Although the expression of BMP genes have been studied in several fish species, our understanding on these genes are limited to their discrepant expression at different developmental stage but not on the change body structure and function (Myers et al. 2002; Marques et al. 2014; Palomino et al. 2014; Tiago et al. 2014; Marques et al. 2015). In marine fish, the expression of BMP genes has been studied when fish larvae are under different supply of nutrients such as vitamins and lipids (Villeneuve et al. 2005a, b, 2006). Recently, BMP genes have been used to evaluate the hyperthermic effects on the skeletal malformation of fish larvae (Ytteborg et al. 2010). Up to present, information on the expression of BMPs in golden pompano is rare despite a high frequency of jaw deformation during the period of larval fish development. Investigation on the expression of BMPs in the ontogeny of golden pompano may provide a hint on the reason associated with jaw malformation in fish larvae during osteogenesis.

Golden pompano belongs to the Carangidae family and is a potential species for aquaculture diversification (Guo et al. 2014). Although the early ontogenetic development of digestive functionality (Ma et al. 2014a, b) and weaning strategies have been studied on golden pompano (Ma et al. 2014d), high jaw malformation during the early developmental stages has severely compromised production efficiency of this fish species in hatcheries. Our previous studies have identified the type, position, and frequency of jaw and skeletal malformations in golden pompano larvae (Ma et al. 2014c; Zheng et al. 2014), but factors causing skeletal malformation on this fish have never been evaluated. This paper aims to understand the impact of water temperature on jaw malformation of golden pompano larvae from the perspective of BMP expression on 18 DPH when jaw deformity starts to occur when weaning starts. The results of this study may contribute to improvement of fish quality and production efficiency in farming golden pompano and other marine fish larvae.

## Methods

Fertilized eggs from the same brood cohort were obtained from a fish farm in Lingshui, Hainan Province, and transported to the Tropical Fisheries Research and Development Center, South China Sea Fisheries Research Institute, Chinese Academy of Fishery Science, Xincun Town. Upon arrival, all eggs were transferred into 500-L incubators until hatching. The water temperature was maintained at 26 °C in the incubators. The experimental design included three constant temperatures 26, 29, and 33 °C with three replicates each. On 2 days post hatch (DPH), yolk sac larvae were acclimatized at each desired temperature for 5 h, and then stocked in 500-L fiberglass tanks at a density of 60 fish L^−1^. All rearing tanks were supplied with filtered seawater with a 5-µm filter from the bottom of each tank through upwelling with a daily water exchange rate of 200 % tank volume. One air stone was used in each tank to maintain dissolved oxygen close to saturation (6.72 ± 0.21 mg L^−1^) and also to promote even distribution of microalgae, rotifers and *Artemia* nauplii. Light intensity was maintained at 2300 lux (measured at the surface) at the light phase under a photoperiod of 13L:11D. Salinity was maintained at 33 ‰ throughout the experiment. For each temperature treatment, there were three biological replicates, and nine tanks were used. The experiments were conducted in accordance with the guideline and approved by the Ethics Committee of South China Sea Fisheries Research Institute, Chinese Academy of Fishery Sciences (2014YJ01).

### Feeding protocol

Rotifers (*Brachionus rotundiformis*) were provided to fish larvae from 2 DPH until 13 DPH three times a day at a density of 10 rotifers mL^−1^. Rotifers were cultured at 25.5 °C. The rotifers fed microalgae (*Nannochloropsis* sp.) were enriched with DHA Protein Selco (INVE Aquaculture, Salt Lake City, UT, USA) before the rotifers were added into larval rearing tanks. The enrichment process was followed by the manufacturer’s instruction. Instant microalgae paste (*Nannochloropsis* sp., Qingdao Hong Bang Biological Technology Co., Ltd, Qingdao, China) was also added into larval fish tanks to feed rotifers and also create a green water background for fish larvae. *Artemia* nauplii enriched with DHA Protein Selco (INVE Aquaculture) were introduced to the fish tank from 9 to 18 DPH at 5 nauplii mL^−1^.

### Growth and survival measurement

In each tank, 10 fish were sampled for size measurements during 1, 3, 5, 9, 12 and 18 DPH. Fish were anaesthetized with AQUI-S (New Zealand Ltd., Lower Hutt, New Zealand) and were measured on a stereo microscope (Phenix Optical Instrument Group Co., LTD, China) with an eyepiece micrometer at 10× magnification to the nearest 0.01 mm. Growth was determined by the specific growth rate (SGR) as  %/day using the following equation (Hopkins 1992): SGR = 100 (*LnSL*_*f*_ – *LnSL*_*i*_)/Δ*t*, where *SL*_*f*_ and *SL*_*i*_ were the final and initial fish standard lengths (mm), respectively, and Δ*t* was the time between sampling intervals. At the end of this experiment, fish from each rearing tanks were harvested and counted for the final survival. Degree-days (D°) was calculated using following equation: D^o^ = Δt × T, where T was the rearing temperature (°C) and Δt is the period of time in days.

### Jaw malformation

At the end of this experiment, 100 fish larvae were randomly collected from each rearing tank to examine the incidence of malformation. Fish were anaesthetized with overdosed Aqui-S (AQUI-S New Zealand Ltd., Lower Hutt, New Zealand) and fixed in 10 % neutrally buffered formalin. Jaw deformity was directly assessed on a stereo microscope (Olympus SZ40, Tokyo, Japan) using the criteria described by Ma et al. (2014c). Jaw malformation (%) was calculated by the following equation: Jaw malformation = (malformed larvae/total larvae) × 100.

### Total RNA extraction and reverse transcription

Approximately 50 individuals were collected from each rearing tank on 18 DPH. Total RNA was extracted using TRIzol Reagent (Invitrogen, USA) according to the manufacture protocol. RNA integrity was verified by electrophoresis on a formaldehyde-agarose gel (1.2 %). The RNA concentration was measured by absorbance at 260 nm and the purity was determined at the OD 260/280 ratio (1.7 < OD260/OD280 < 2.0), OD 260/230 ratio (2.0 < OD260/OD230 < 2.5) and agarose gel electrophoresis. RNA was reverse-transcribed to cDNA with oligo (dT) primers using a PrimeScript 1st strand cDNA synthesis kit (TaKaRa Biotechnology, Dalian Co., Ltd). The cDNA was used as a template in subsequent PCR. The cDNAs for quantitative real-time PCR were synthesized from one microgram of the total RNA of each sample using the PrimeScript™ RT reagent kit with gDNA Eraser (TaKaRa).

### Gene cloning

Based on unpublished golden pompano transcriptome sequences (Illumina HiSeq 2000, annotated by NR, KOG, kegg, and Swissprot), the genes cloning primers were designed (shown in Table 1). The reagents for PCR reaction included 1 μL of golden pompano larval cDNA, 1 μL of gene-specific forward primer (F, 10 μmol L^−1^), 1 μL of gene-specific reverse primer (R, 10 μmol L^−1^), 0.5 μL of ExTaq, 5 μL of PCR buffer and 4 μL of dNTP mixture (2.5 μM), 37.5 μL of ddH_2_O in a total volume of 50 μL. The PCR conditions were as follows: denaturation at 94 °C for 1 min, 35-cycles of 94 °C for 30 s, annealing temperature of each genes for 30 s, 72 °C for 4 min, followed by a 10 min extension at 72 °C. The PCR products were cloned into the PMD-19T vector (TaKaRa Biotechnology, Dalian Co., Ltd), and then were sequenced.

**Table 1:** Summary of genes cloning primers used in this study

Primers	Sequence (5′–3′)	Amplicon sizes (bp)
BMP2-F	CGTGCTGACCAAGACCTAAC	
BMP2-R	AACCGGGTGTCCATAATAAC	1549
BMP4-F	GACACCTTCCCTTTCACAT	
BMP4-R	CAAGTCCAAGTTCTAGTTAGTTT	1425
BMP5-F	CCAACGAAGACACTACAAGG	
BMP5-R	TTAAAGTTAGCCCAGCCACT	871
BMP10-F	GAAGGACAGTCCTCCCTCAA	
BMP10-R	TGCAGCATTGCTTTGCTTTA	1854
EF-1α-F	TGTTACCTGGCTAGGGG	
EF-1α-R	GAGAAGAGGCACCGTCA	1662

Note for BMP names: *F* forward primer, *R* reverse primer

### Gene expression analysis by quantitative real-time PCR

Quantitative real-time PCR (qPCR) was used to analyze the expression levels of BMP genes in golden pompano larvae. Gene specific primer pairs for BMP genes (Table 2) were amplified in LightCycler480 II (Roche, Switzerland). The EF-1α (GenBank Accession NO. KT727924) was used as the reference and amplified. The cycling conditions for the BMP genes and EF1α were as follows: 1 min at 95 °C, followed by 40 cycles at 95 °C for 15 s, and 60 °C for 1 min. Dissociation curves were employed to ensure that only one single PCR product was amplified in each gene reaction. For each test, three replicates were performed. The relative quantification (RQ) was calculated using the ^ΔΔ^CT (comparative threshold cycle) method (^Δ^CT = CT of target gene − CT of EF-1α, ^ΔΔ^CT = ^Δ^CT of any sample − ^Δ^CT of calibrator sample). The efficiencies of the primers (E) were E_BMP2_ = 0.998, E_BMP4_ = 1.004, E_BMP5_ = 0.923, E_BMP10_ = 1.004.

**Table 2:** Summary of quantitative real-time PCR primers used in this study

Primers	Sequence (5′–3′)	Amplicon sizes (bp)
BMP2-qF	CAGGCAGCACTCCGCAAAC	
BMP2-qR	TCCCCGTGGCAGTAAAAGG	146
BMP4-qF	GTGAACAACAACATTCCCAAGG	
BMP4-qR	GCAGCCCTCCACTACCATTT	126
BMP5-qF	GTGGAGACTGTAGACGGACGAA	
BMP5-qR	TGAAGAAAGCAACCAGGAAGG	100
BMP10-qF	CCGCTTCAGTCTTCTCCAACC	
BMP10-qR	CGGATTATCACCCACATCCCTA	149
EF-1α-qF	CCCCTTGGTCGTTTTGCC	
EF-1α-qR	GCCTTGGTTGTCTTTCCGCTA	101

Note for BMP names: *qF* forward primer for real-time PCR, *qR* reverse primer for real time PCR

### Statistical analysis

All percentage data were arcsine-transformed prior to statistical analysis. However, the data were presented as untransformed values in figures. The data were all expressed as mean ± SD, and compared with one way ANOVA (PASW Statistics 18.0, Chicago, SPSS Inc.). When a significant treatment effect was found, Tukey’s test was performed for multiple range comparisons with the level of significant difference set at *P* < 0.05. All data were tested for normality, homogeneity and independence to satisfy the assumptions of ANOVA.

## Results

### Growth performance, survival and jaw deformities

**Fig. 1:**
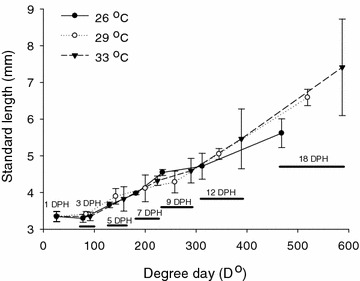
Standard length of golden pompano larvae cultured at 26, 29, and 33 °C from 1 day post hatching (DPH) to 18 DPH

At the end of this experiment, the standard length of fish larvae at 26, 29, and 33 °C were 5.62 ± 0.39, 6.59 ± 0.22, and 7.41 ± 1.31 mm, respectively. Temperature significantly affected the growth of golden pompano larvae (*P* < 0.05, Table 3). The SGRs of fish at 29 and 33 °C were 3.64 ± 0.20 and 4.31 ± 0.74 %/day, respectively, which were significantly higher than those reared at 26 °C (*P* < 0.05). The SGR of fish was not significantly different between fish at 29 and 33 °C (*P* > 0.05). During the ontogenetic development, the variation of fish length on each sampling day increased with the increase of degree-days (Fig. 1). In this study, the growth of newly hatched golden pompano was slow before 234 degree-day (D°) on 9 DPH. When the number of degree-days reached 312–389 D° (12 DPH), the size of fish larvae gradually became different between temperature treatments. When the degree-days reached 468–587 D° (18 DPH), fish growth was clearly affected by water temperature, and fish growth was accelerated when temperature increased from 29 to 33 °C.

**Table 3:** Specific growth rate, survival, and jaw deformity rate of golden pompano larvae cultured at 26, 29, and 33 °C

	26 °C	29 °C	33 °C
Specific growth rate (%/day)	2.70 ± 0.42^a^	3.64 ± 0.20^b^	4.31 ± 0.74^b^
Survival (%)	10.30 ± 0.41^a, b^	11.36 ± 1.08^b^	9.57 ± 0.23^a^
Jaw deformity rate (%)	5.00 ± 0.64^a^	10 ± 2.65^b^	20.00 ± 3.54^c^

Different lowercase letters (i.e., a, b and c) indicate statistically significant differences (*P* < 0.05

Rearing temperature significantly affected the survival of golden pompano larvae (*P* < 0.05, Table 3). The highest survival was achieved in fish at 29 °C, and the lowest survival was observed in fish at 33 °C. In this study, water temperature significantly affected fish jaw deformities (*P* < 0.05, Table 3). Jaw deformity of fish at 33 °C was 20.00 ± 3.54 %, which was significantly higher than the deformity rates of fish at 26 and 29 °C (*P* < 0.05). The lowest jaw deformity rate was observed in fish reared at 26 °C.

### Expression of BMPs in fish at different temperatures

**Fig. 2:**
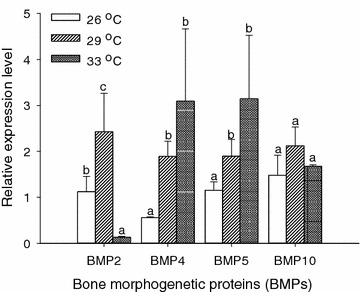
Relative expression levels of bone morphogenetic proteins of golden pompano larvae at different temperatures on 18 DPH. The level of gene expression at 26 °C was served as the control for BMP2, BMP4, BMP5, and BMP10, respectively

Partial sequences of BMP2 (GenBank Accession NO. KT727918), BMP4 (GenBank Accession NO. KT727919), BMP5 (GenBank Accession NO. KT727921), and BMP10 (GenBank Accession NO. KT727920) genes were obtained after sequencing analysis (Appendices 1–4). Water temperature significantly affected the expressions of BMP2, BMP4 and BMP5 genes in golden pompano larvae (*P* < 0.05, Fig. 2). In the gene expression of BMP2, the highest expression was found in fish at 29 °C, and the lowest expression was in fish at 33 °C. In the gene expressions of BMP4 and BMP5, the expression levels were not significantly different between fish at 29 and 33 °C (*P* > 0.05), but were significantly higher than fish reared at 26 °C (*P* < 0.05, Fig. 2). On 18 DPH, the expression of BMP10 in golden pompano larvae was not significantly affected by the rearing temperature (*P* > 0.05).

## Discussion

### Growth and survival of fish larvae

Water temperature is a critical factor for the success of fish hatchery production, and it has significant implication on the growth performance of fish larvae (Boglione et al. 2013b). Temperature can regulate fish metabolism, food intake and growth (Jobling 1994; Ma 2014), and the effects of temperature on fish growth have been well documented in the larvae of commercially important fish species including haddock *Melanogrammus aeglefinus* L. (Martell et al. 2005), nase *Chondrostoma nasus* L. (Keckeis et al. 2001), Australian snapper *Pagrus auratus* (Fielder et al. 2005), striped trumpeter *Latris lineata* (Choa et al. 2010), and yellowtail kingfish *Seriola lalandi* (Ma 2014). In golden pompano, the fast growth at high temperature may be attributed to the improved food ingestion and digestive function of fish larvae after 15 DPH as Ma et al. (2014b) reported that the goblet cells and gastric glands appeared in the gut of golden pompano larvae after 15 DPH at 27–29 °C. The growth of fish larvae tended to be accelerated when fish were weaned from rotifers to *Artemia* nauplii. Similar to the Florida pompano *Trachinotus* carolinus (Riley et al. 2009), the length of mouth gape close to 1.05 mm should allow golden pompano larvae to ingest *Artemia* nauplii and other similar size of food particles by 12 DPH. Therefore, the significant difference in fish size between thermal treatments at 18 DPH may be also related to the use of high energy food from 9 DPH onwards.

In both wild and artificial environments, fish mortality is often observed at the critical period of nutritional transition from endogenous to exogenous feeding (Otterlei et al. 1999; Ma et al. 2012). During the period of food transition, when the food supply and light condition are optimal, temperature can be a key determinant for fish survival (McGurk 1984; Kamler 1992; Gardeur et al. 2007; Ma 2014). Previous studies have demonstrated that mortality is strongly temperature-dependent in the larvae and juveniles of *Seriola lalandi* (Ma 2014), *Pangasianodon hypophthalmus* (Baras et al. 2011), *Inimicus japonicas* (Wen et al. 2013), and *Glyptocephalus cynoglossus* (Bidwell and Howell 2001). Ma (2014) suggests that there is a temperature-sensitive period during early ontogeny where mortality is likely to occur in fish larvae. In the present study, lower survival was observed when fish were reared at 33 °C than at other temperatures, suggesting the importance of temperature for golden pompano larval rearing. In the present study, the fish density was reduced at high temperatures due to high mortality, which may contribute to temperature-dependent fish growth in this study.

### Temperature effect on jaw malformation

Jaw malformation is a major concern in fish culture because it affects fish morphology and quality at the growout stage (Von Westernhagen 1988). In the present study, the proportion of fish larvae exhibiting jaw deformities increased with the increase of water temperature, and peaked at 33 °C. Similar results have also been found in other fish species such as Atlantic halibut *Hippoglossus hippoglossus* (Lein et al. 1997) and Pacific herring *Clupea pallasi* (Alderdice and Velsen 1971). Such a temperature-dependent developmental pattern is generally attributed to high oxygen (Rombough 1997) and high nutritional requirements at high temperatures, which may not be fulfilled unless the feed with high energy or protein contents is provided (Cahu et al. 2003a, b; Ma 2014). Furthemore, temperature fluctuation might also interfere with the harmonic development of functional organs such as the digestive tract and skeleton, leading to skeletal deformities at high temperature. In the present study, fertilized fish eggs hatched at 26 °C, and yolk sac larvae were acclimatized at each desired temperature for 5 h on 2 DPH. The quick increase of rearing temperature from 26 to 29 or 33 °C may affect skeletogenesis and induce jaw deformity.

### BMP gene expression at different temperatures

The present study was design to evaluate the effects of temperature appear to be specific to certain BMP family members impacting bone formation. The growth of skeleton relies on the dynamic equilibrium between cartilage production and bone apposition rate (Breur et al. 1991). The BMP2 and BMP4 proteins are involved in processes of dorsal–ventral axis specification (Graff 1997), epithelio-mesenchymal interactions (Vainio et al. 1993), and apoptosis (Graham et al. 1994; Glozak and Rogers 1996; Zou and Niswander 1996). The BMP2 gene in zebrafish is related to the induction and maintenance of ventro-lateral cell fate during early development, while a missense mutation in the BMP2b gene can lead to the early dorsalized phenotype of the zebrafish *swirl* mutant which lacks the cardiogenic mesoderm (Kishimoto et al. 1997). Ytteborg et al. (2010) found that the expression of BMP2 was up-regulated when fish was under a hyperthermic condition. In the present study, the expression of BMP2 in fish reared at 29 °C showed a up-regulating trend comparing to the expreesion of BMP2 in fish reared at 26 °C, which is consistent with the results reported by Ytteborg et al. (2010). However, the reason for low expression of BMP2 in fish at 33 °C is unclear.

BMP4 plays a diverse role during vertebrate development (Hogan 1996b; Mehler et al. 1997; Whitman 1998; Dale and Johns 1999; Shi and Massague 2003), and has been used to evaluate if the BMP metabolic pathway is related to skeletal deformities under an unbalanced nutritional supply (Villeneuve et al. 2005a, b, 2006) (Ytteborg et al. 2010). Villeneuve et al. (2006) suggested that the increase of BMP4 and RARγ expression can reduce the number of osteoblasts available for bone formation and that the loss of bone cells is counterbalanced by the cooperation between retinoic acid and BMP4. In the present study, the expression of BMP4 in fish at 29 and 33 °C increased significantly, and jaw deformities at these two temperatures were significantly higher than fish at 26 °C. This result supports to the notion that the expression of BMP4 tends to increase when fish are reared at a high temperature (Ytteborg et al. 2010), and the proportion of jaw deformities in fish larvae increases when the expression of BMP4 in fish shows a trend of up-regulation (Villeneuve et al. 2006). However, to confirm this result, future research should evaluate the gene expression in the jaw and conduct in situ hybridization analysis and mineralization analysis.

Previous studies indicate that the 60A subgroup (BMPs5, 6 and 7) is functionally redundant and that the collective expression of the 60A subgroup determines developmental function (Solloway and Robertson 1999; Kim et al. 2001). Specifically, BMP5 can be expressed during endochondral ossification, inducing condensation of mesenchymal cells to chondrocytes (King et al. 1994; Bailon-Plaza et al. 1999). Furthermore, the mutated BMP5 gene can lead to skeletal and bone abnormalities, suggesting the importance of BMP5 in skeletal development (Kingsley et al. 1992; Storm et al. 1994; Wolfman et al. 2003). In the present study, the expression pattern of BMP5 was similar to that observed in BMP4. The expression level of BMP5 in fish at 29 or 33 °C was significantly higher than that at 26 °C. Although the expression of BMP5 increased with increasing temperatures and the occurrence of jaw deformity, it is unclear if deformity and high temperature are concomitant events in fish.

Existing literature indicates that the BMP10 gene plays little role in craniofacial or bone development. The BMP10 gene is expressed predominantly in the adult heart and to a lesser extent in the liver and lung (Neuhaus et al. 1999). During heart development, BMP10 is expressed in the trabeculae of the *bulbus cordis*, the common ventricular chamber, and of the atrium (Neuhaus et al. 1999). In zebrafish, relatively high BMP10 expression occurs in the heart and liver, but low expression is detected in the brain, and kidney (Bland 2001). In the present study, the expression of BMP10 was not significantly affected by the rearing temperature. This may suggest that the expression of BMP10 in golden pompano larvae is not sensitive to temperature by 18 DPH.

## Conclusion

In summary, the present study examined the effect of temperature on the jaw deformity in golden pompano larvae. Jaw deformity in fish larvae increased with the increase of water temperature, and peaked at 33 °C. This study suggests that the rearing temperature of golden pompano larvae should be controlled at 26–29 °C and the expression levels of BMP4 and BMP5 genes are positively synchronized with the occurrence of jaw deformities.
